# A bond valence sum method to identify potential new fluoride ion conductors

**DOI:** 10.1107/S1600576725009574

**Published:** 2025-11-28

**Authors:** Robbie Shaw, Gabriel E. Pérez, Helen Playford, Stephen Hull

**Affiliations:** a ISIS Neutron and Muon Source, Science and Technology Facilities Council, Rutherford Appleton Laboratory, Harwell Campus, Didcot OX11 0QX, United Kingdom; b EaStCHEM, School of Chemistry, University of St Andrews, St Andrews KY16 9ST, UK; University of Tennessee, USA; Oak Ridge National Laboratory, USA

**Keywords:** fluoride conductors, bond valence sums, lone-pair electrons, ionic diffusion, materials discovery

## Abstract

The bond valence sum (BVS) method has been extensively used to probe the ionic diffusion pathways within solids possessing high ionic conductivities, both anionic and cationic. For the former, the presence of lone-pair cations such as Tl^+^, Sn^2+^, Pb^2+^, Sb^3+^ and Bi^3+^ is known to enhance the anionic conductivity of solids by promoting disorder within the anion substructure and lowering the energy barriers between lattice sites. However, the BVS formalism assumes spherical ions and does not include the possibility of the more irregular co­ordination environments associated with electron lone pairs. We present here a simple modification of the BVS approach, which has been implemented in the form of a Python program that permits the preferred F^−^ diffusion pathways to be determined in fluoride ion conductors containing lone-pair cations. The method has been benchmarked by calculating energy barriers that broadly correlate with the F^−^ ionic conductivities of known compounds, and successfully reproduces the F^−^ distribution within the highly conducting compound β-PbSnF_4_ determined by neutron powder diffraction and molecular dynamics (MD) methods. Using this new approach, fluoride compounds within the Inorganic Crystal Structure Database that contain at least one lone-pair cation have been screened and several new potential F^−^ ion conductors have been identified.

## Abstract 2

A simple modification to the bond valence site energy method is proposed to account for the asymmetrical coordination around post-transition metal cations in fluoride conductors due to the presence of electron lone pairs. A total of 136 structures from the Inorganic Crystal Structure Database are screened and several potential new conductors are suggested.

## 1. Introduction

To prepare for the upcoming energy transition to renewable technologies, significant research has been dedicated to finding alternative battery chemistries to lithium. Much attention has been focused on developing batteries that use other mobile cations, such as the monovalent sodium ion (Usiskin *et al.*, 2021 ▸) and multivalent ions including divalent magnesium and trivalent aluminium (Liang *et al.*, 2020 ▸). In addition, the use of diffusing anions in batteries has been investigated. Interest in fluoride ion batteries began in earnest in 2011, when Anji Reddy & Fichtner (2011 ▸) reported a reversible all-solid-state battery. Fluoride ion batteries have several promising characteristics, such as high volumetric and gravimetric energy densities and a high open-circuit voltage (Nowroozi *et al.*, 2021 ▸). However, the lack of suitable electrode and electrolyte materials with good ionic conductivity is currently the main barrier towards the adoption of fluoride ion batteries as a viable energy storage technology (Anji Reddy & Fichtner, 2011 ▸). In this paper, we present a modification to the bond valence site energy screening technique, with the aim of discovering promising fluoride ion conductors for use as solid electrolytes in future fluoride ion batteries.

## 2. Fluoride ion conductors

High fluoride ion conductivity at elevated temperatures is known to occur in many compounds, including many adopting tysonite, perovskite and fluorite crystal structures [for comprehensive reviews, see Anji Reddy & Fichtner (2016 ▸) and Gopinadh *et al.* (2022 ▸)]. The fluorite structure can be described as a primitive substructure of fluoride ions, with cations occupying alternate cube centres. This arrangement is adopted by the mineral fluorite (CaF_2_) and several other binary halides, with these compounds typically showing a type-II superionic transition at a temperature *T*_c_ around 0.6–0.8*T*_m_, where *T*_m_ is the melting temperature in kelvin [for a review, see Hull (2004 ▸)].

Particular attention has focused on the fluorite polymorph of lead fluoride, named β-PbF_2_, since it has the lowest superionic transition temperature [*T*_c_ = 711 K; Schroter & Nolting (1980 ▸)]. However, its ionic conductivity at ambient temperature is too low to be used in fluoride ion batteries, and aliovalent cation doping has been extensively used to increase the F^−^ ion conductivity of β-PbF_2_ at temperatures close to ambient. For example, Hull *et al.* (1998 ▸) showed that the doping of β-PbF_2_ with KF increases the ionic conductivity of the pure material at 350 K by three orders of magnitude via the formation of charge-compensating anion vacancies.

High F^−^ ion conductivity at room temperature also occurs in ordered compounds based on PbF_2_, with the fluorite-related PbSnF_4_ showing a conductivity of 10^−3^ to 10^−2^ S cm^−1^ under ambient conditions (Réau *et al.*, 1978 ▸; Castiglione *et al.*, 2005 ▸). A distinctive feature of PbSnF_4_ is the stereoactivity of the Sn^2+^ ions, which adopt a square-pyramidal coordination instead of the symmetric cubic coordination shown by the Pb^2+^ ions. This second-order Jahn–Teller effect leads to the displacement of fluoride ions off their normal cubic site onto an interstitial site, forming a more disordered arrangement of the anions. This effect is often referred to as a lone-pair effect, where the filled 5*s* orbital has hybridized with a 5*p* orbital and become directional. In a study of post-transition metal oxides by Walsh *et al.* (2011 ▸), it was found that these are not in fact lone pairs, although there is an increase in electron density at the traditional lone-pair position in the highest occupied molecular orbital (HOMO). For ease, this is still referred to as a lone-pair effect in the remainder of this paper. The net result is the enhancement of the F^−^ conductivity by adding disorder and providing a variety of hopping pathways within the structure. This is discussed further in Section 8.1 (Castiglione *et al.*, 2005 ▸; Murray *et al.*, 2008 ▸).

Beyond the example of PbSnF_4_, a number of compounds containing at least one lone-pair cation such as Tl^+^, Sn^2+^, Pb^2+^ and Bi^3+^ show high F^−^ ion conductivity, at least at elevated temperatures, including TlBiF_4_ (Lucat *et al.*, 1977 ▸), Cs_2_SnF_5_ (Berastegui *et al.*, 2010 ▸) and KPbF_3_ (Hull *et al.*, 1998 ▸). Thus, our approach to identifying new F^−^ ion conductors focuses on these cations and incorporates the effects of asymmetrical coordination into the screening process.

## 3. Materials screening

In the past, the discovery of new ionic conductors was largely driven by chemical intuition or chance. However, computational screening is now a powerful approach, aided by recent vast increases in computational power and the availability of crystallographic databases such as the Inorganic Crystal Structure Database (ICSD; Zagorac *et al.*, 2019 ▸), the Crystallographic Open Database (Gražulis *et al.*, 2009 ▸) and Materials Project (Jain *et al.*, 2013 ▸). In this work, the ICSD is used as the source of crystallographic data, as it contains the largest number of candidate structures.

When screening for new ionically conducting materials, there is an inherent trade-off between accuracy and speed. Molecular dynamics (MD) simulations have been used extensively to model ionic conduction processes [see, for example, Castiglione *et al.* (2005 ▸) for the case of PbSnF_4_]. These studies may use classical force fields or reach a higher level of sophistication using density functional theory. With the increase in computational power available, recent studies have successfully used MD to screen a large number of compounds (Kahle *et al.*, 2020 ▸). Conduction barriers can also be estimated using nudged elastic band methods, including that introduced by Sundberg *et al.* (2022 ▸) for fluoride ion conductors.

Despite the comments above, simpler screening methods have a number of advantages, being computationally cheaper, more sustainable and more accessible to a wider range of users. In addition, the increasing number of predicted structures may surpass the capacity for more sophisticated techniques. Both Voronoi geometric analysis and bond valence techniques have been utilized *en masse* to screen 29000 structures, including fluoride conductors, by Zhang *et al.* (2020 ▸). However, their approach did not take into account lone-pair effects which, as discussed above, often have a profound influence on the ionic conduction processes.

## 4. The bond valence model

In the bond valence model, a crystalline material is described as a network of atoms where pairs of adjacent atoms of opposite charge are connected by a bond. Each bond can be described with a valence *s* derived from its experimental bond length *r*_*ij*_ and two empirical parameters *r*_0_ and *b*,
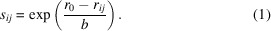
These empirical parameters have been comprehensively tabulated and can be applied to almost all crystal structures (Adams & Rao, 2014 ▸; Brown, 2016 ▸). Originally, when only the first coordination shell was considered, *r*_0_ and *b* could not reliably be determined independently, and hence *b* was set to a fixed value of 0.37 Å. However, by including contributions from the higher coordination shells, both parameters can now be determined reliably (Adams, 2001 ▸).

For the calculation of conduction routes, parameter sets with varying *b* values are recommended as they take into account the weak interactions with atoms in the second coordination shell. As such, the *softBV* parameter set (Chen *et al.*, 2019 ▸) is used in the following calculations. Each atom adheres to the valence sum rule as stated by Brown (2016 ▸): ‘the sum of experimental valences of all bonds formed by an atom is equal to the valence of an atom.’ Waltersson (1978 ▸) and Garrett *et al.* (1982 ▸) used this idea to develop the concept of bond valence maps, which can be used to model ionic diffusion. For any point in the crystal structure, the bond valence sum mismatch (BVSM) compares the difference between the ideal valence for that ion and the valence it would have if located on that site:
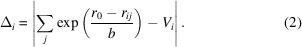
By proceeding stepwise through the crystal structure, a map can be created showing pathways of low mismatch. These paths represent positions where ions should be more stable, and hence they indicate possible conduction routes through the structure. This approach has been used to evaluate possible conduction paths of Ag^+^ and Li^+^ conductors (Adams & Maier, 1998 ▸; Adams, 2006 ▸).

## 5. Bond valence site energy

In an extension to the above, it is possible to calculate energy landscapes using a potential derived from the bond valence (Adams & Rao, 2009 ▸; Chen *et al.*, 2019 ▸). This bond valence site energy (BVSE) pseudopotential contains two contributions, a Morse-type term that describes the attractive and Born repulsion interaction between cation–anion pairs, and a Coulombic repulsion term between cation–cation and anion–anion pairs. The Morse potential is calculated from three parameters, the bond breaking energy *D*_0_, the equilibrium bond distance *r*_min_ and the bond softness parameter *b*:
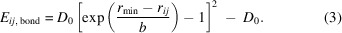
The terms *D*_0_ and *r*_min_ can be calculated from the original bond valence parameters (Chen *et al.*, 2019 ▸). In the original remit of the bond valence method, the Coulomb repulsions are not of great importance. However, when considering conduction pathways and the creation of a pseudopotential, it is essential to include the effects of repulsion. Therefore, a Coulombic repulsion term is added of the form
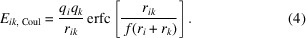
Here, *q*_*i*_ is the effective charge of the ion, erfc() signifies the complementary error function, *r*_*i*_ is the covalent radius of the atom and *f* is a screening factor. Combining these two terms, an energy landscape can be generated which can be subsequently analysed to find conduction paths and their energy barriers. BVSE psuedopotentials have been widely used to analyse conduction and as a high-throughput search tool for many different ions (Kabanov *et al.*, 2024 ▸; Zhang *et al.*, 2020 ▸; Kabanova *et al.*, 2024 ▸).

## 6. Lone-pair modelling

The effect of lone pairs on bond valence has been discussed previously by Wang & Liebau (2007 ▸), who found the bond valence sum to be altered by the coordination environment’s level of asymmetry. In the context of the present study, the most important issue is preventing the occupation of sites due to the presence of electron lone pairs. In order to achieve this, dummy lone pairs are placed into the crystal structure, repelling nearby fluoride ions.

In order to model the effect of these lone pairs, their location and directionality must first be found. For the current study, five main group cations (Sn^2+^, Sb^3+^, Tl^+^, Pb^2+^ and Bi^3+^) were considered as having potential for stereoactivity. Given a crystal structure, we first test these cations for an asymmetric coordination environment. This is achieved through calculating the atoms’ bond valence vector sum 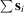, where each bond valence contribution is multiplied by a unit vector representing the bond’s direction:
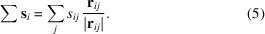
For a symmetrically coordinated ion, the vector sum should equal zero (Harvey *et al.*, 2006 ▸). Otherwise, the vector sum will indicate some degree of asymmetry in the coordination. If the magnitude of the vector sum is above an arbitrary cutoff (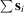
 > 0.5 units was found to work well), a dummy lone-pair site is then added to the structure at a distance of 1 Å in a direction opposite to the bond valence unit vector. A distance of 1 Å for a hypothetical Sn lone-pair bond has been suggested previously (Galy *et al.*, 1975 ▸; Brown, 2009 ▸), and testing showed the calculated conduction barriers only varied by a maximum of 0.01 arbitrary units upon a 0.5 Å change in the lone-pair distance.

Once these dummy lone-pair sites have been established, the bond valence calculations can proceed. Implementation of the lone-pair sites was trialled in both the BVSM calculation and the BVSE calculation. To implement it in the BVSM calculation, a simple penalty function *p*(*r*) is added to the bond valence mismatch Δ_*i*_ to avoid anion occupancy of sites close to the lone pair, as shown in equation (6):
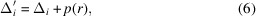
Both a linear and a quadratic function were trialled. The linear function [equation (7)] resembles the radial dependence of the Coulombic potential between two point charges, whereas the quadratic function [equation (8)] resembles the radial dependence of the Coulombic potential between a point charge and a dipole:
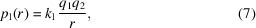

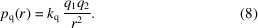
The penalty function constants *k* were visually refined by comparing the resulting conduction maps with neutron data and MD simulations for PbSnF_4_ (Castiglione *et al.*, 2005 ▸). Comparison with these data showed that better results were obtained using the quadratic penalty function, as the linear penalty function reduced the occupancy of nearby sites too much.

For the BVSE calculation, the lone-pair penalty could be much more cleanly integrated by inclusion in the repulsion term [see equation (4)]. The lone pairs were assumed to have a charge of −2.

## 7. Implementation

To implement this method, we have developed a program, named *Lone Pair BV*, that can calculate both BVSM and BVSE maps for any ordered crystal structure, though it has only been extensively tested here on fluoride compounds. The program functions by iterating through a set of voxels in the crystal structure, calculating a BVSM/BVSE value at each point. Although not fully optimal, for simplicity the code calculates voxels throughout the whole unit cell and ignores any symmetry elements. Some simplifications were made to the BVSE calculation compared with the previous work of Chen *et al.* (2019 ▸), including a universal screening factor of 0.75 for all structures and the use of formal oxidation states of the ions rather than effective charges. Testing with a variety of fluoride structures showed that the inclusion of these terms made little impact on the produced maps. Hence, for simplicity, these were removed. However, as a result, the calculated conduction barriers can only be compared with results also obtained from the *Lone Pair BV* program.

*Lone Pair BV* was coded in Python and is available on Github (accessible via https://github.com/robbie6shaw/lone-pair-BV). The program currently consists of three Python scripts. The program is launched through run.py, which contains a variety of functions for the user. Normal use of the program consists of the user inputting two commands – the create_input command to convert the .cif file to a custom input file where a unit cell is defined without any symmetry, followed by the bvsm or bvse command to use this input file to produce the map. The bulk_bvse command can also be used to run calculations for a folder of .ciffiles. When these functions are called, run.py calls two other scripts, fileIO.py and bvStructure.py. The fileIO.py script contains methods for communicating with the bond valence database and converting .cif files to input files. The bvStructure.py script contains the BVStructure class, which contains the methods used to create a map from an input file. The *Numba* library (https://numba.pydata.org/) is used to translate the Python code into fast machine code, allowing a reduction in calculation time for a typical structure from approximately 15 min to under 1 s on a modern desktop computer.

During the development process, it became clear that the BVSE maps were superior to the BVSM maps, allowing cleaner integration of the lone pairs and generating maps that better matched experimental ion distributions. This also allowed the use of the migration pathway analyser provided in *softBV* to evaluate the generated maps (Chen *et al.*, 2019 ▸; Wong *et al.*, 2021 ▸). The analyser identifies voxels in the landscape that are local minima or saddle points, connects them, and calculates their barrier. The analyser calculates separate conduction barriers in arbitrary units (a.u.) for each dimension (one-, two- and three-dimensional) and a combination of the barriers was used to evaluate the conduction. Therefore, only BVSE maps are discussed in the remainder of this paper.

## 8. Benchmarking

### 8.1. Fluorine distribution in PbSnF_4_

**Figure 1:**
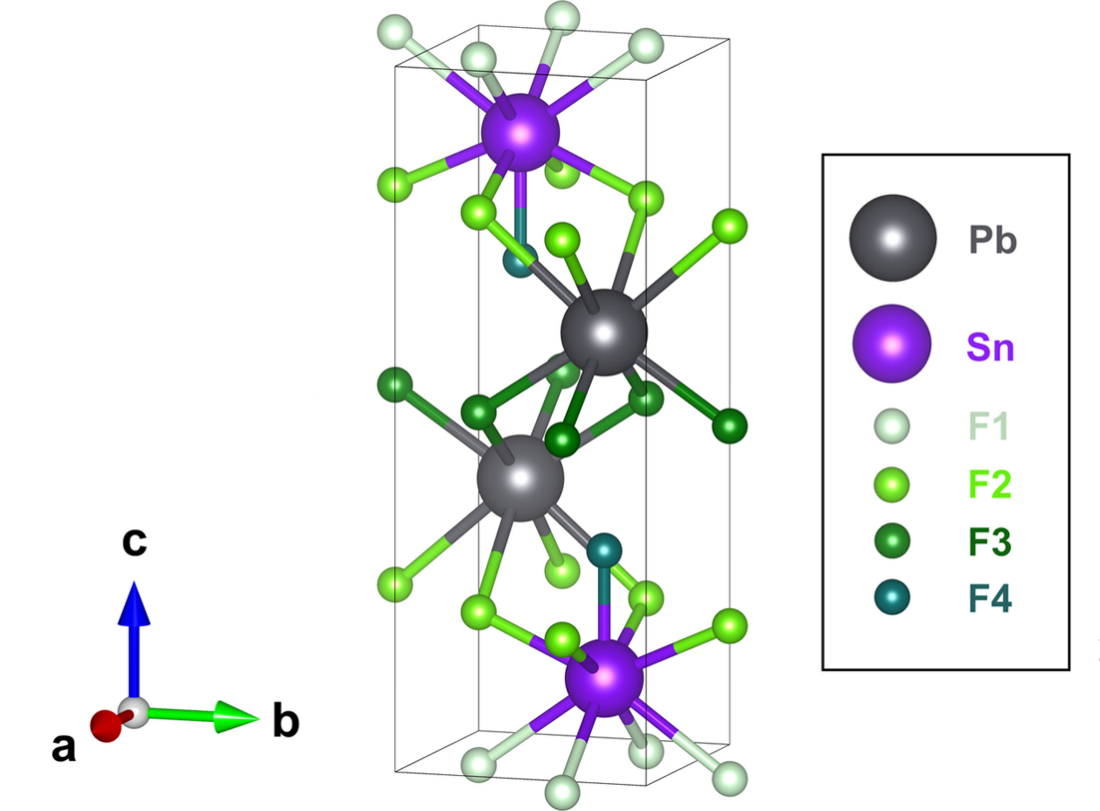
Crystal structure of PbSnF_4_ determined by Castiglione *et al.* (2005 ▸). Pb^2+^, Sn^2+^ and F^−^ sites are labelled in grey, purple and green, respectively. The F1 site (pale green) is unoccupied at room temperature.

An initial assessment of the BVSE map approach outlined above was made using the tetragonal polymorph of the compound PbSnF_4_, chosen because it has a very high F^−^ ion conductivity at ambient temperature and the anion conduction pathways have been characterized by neutron powder diffraction and MD methods (Castiglione *et al.*, 2005 ▸). As shown in Fig. 1 ▸, PbSnF_4_ adopts a tetragonal layered superstructure of the fluorite arrangement, with the two cation species ordered in the sequence PbPbSnSnPbPb… along the *c* axis. The anion sites within the cubic fluorite aristotype are then split into three symmetry-independent sites, F1, F2 and F3, within the SnSn, SnPb and PbPb layers, respectively. The F3 sites in PbSnF_4_ are bound to their ideal sites, making minimal contribution to the overall anion conductivity. Conversely, the F1 sites are empty, as a consequence of lone pairs on Sn^2+^. The displaced anions sit on interstitial (F4) sites within the SnPb layers and, together with anions on the F2 sites, form a highly disordered F^−^ distribution which is responsible for extensive diffusion within those layers in the (001) planes (Castiglione *et al.*, 2005 ▸).

**Figure 2:**
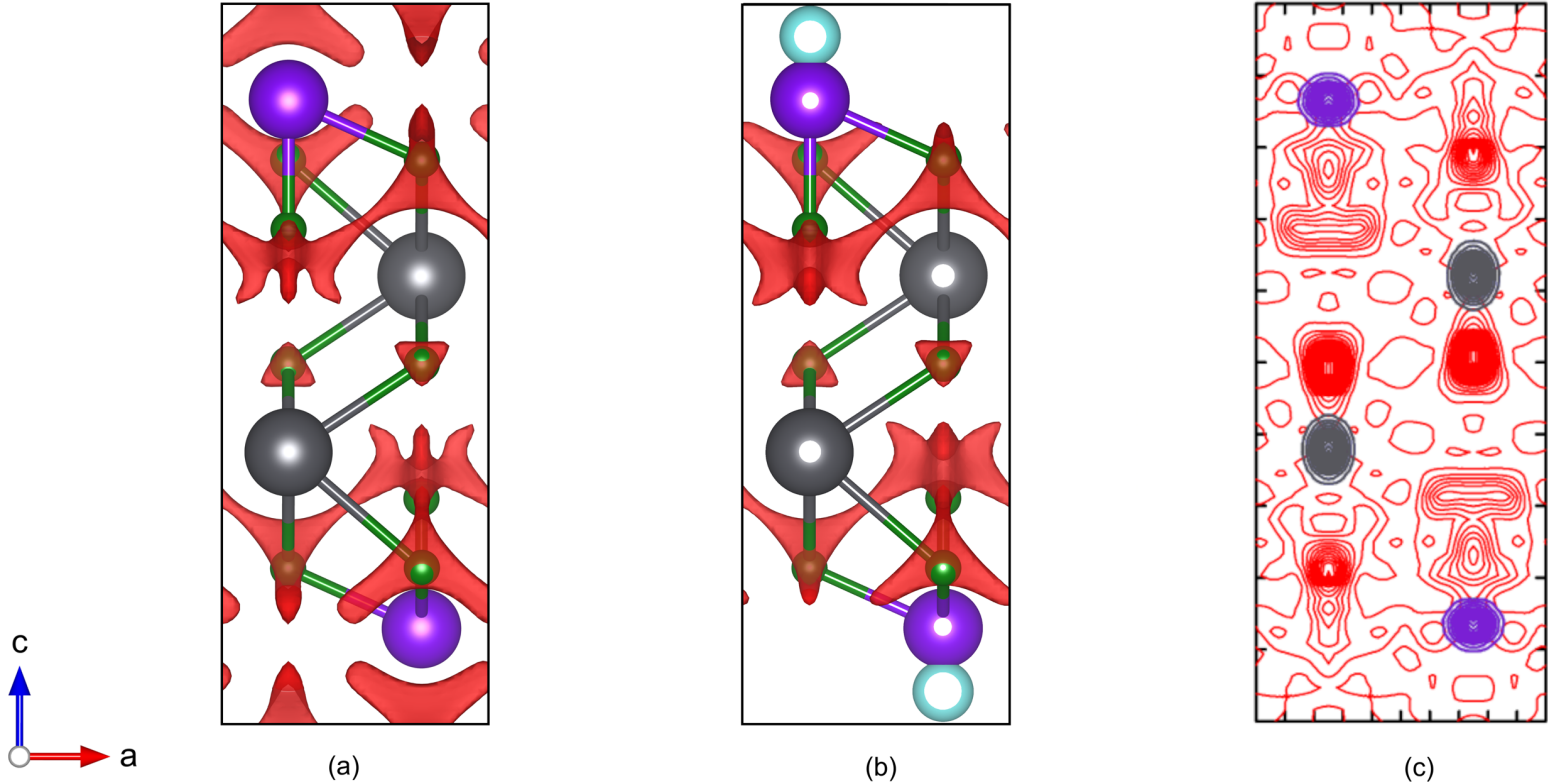
Diagrams of [010] projections of PbSnF_4_, with Pb^2+^ and Sn^2+^ shown as grey and purple spheres, respectively. (*a*) A bond valence site energy map without the lone-pair corrections. The red isosurface bounds energies 0.2 a.u. above the minimum energy. (*b*) A bond valence site energy map with the lone-pair corrections (pale blue spheres). The red isosurface bounds energies 0.2 a.u. above the minimum energy. (*c*) The experimentally determined time-averaged distribution of ions at 298 K determined using neutron diffraction. Structural data and experimental ion distributions from Castiglione *et al.* (2005 ▸).

Fig. 2 ▸ shows a comparison between the time-averaged distribution of ions and the calculated BVSE maps with and without the lone-pair corrections. Use of existing software such as *softBV* would give a conduction map similar to Fig. 2 ▸(*a*), in which the F3 site appears non-conductive as expected and conduction routes between F2 and F4 sites are found. The program correctly predicts that ionic density would be smeared along the *c* axis for the F2 site and perpendicular to the *c* axis for the F4 site. However, the map includes conduction paths within the SnSn layer which are not seen experimentally. This is unsurprising, as the bond valence model assumes each ion has a balanced coordination environ­ment, and it highlights the need to include lone-pair effects to reproduce correctly the conduction routes in these compounds.

Fig. 2 ▸(*b*) shows the map produced with the lone pairs included, using the approach outlined in Section 7 above. The program has correctly placed lone-pair dummy sites on the Sn atoms, pointing into the space between the two Sn layers. After taking these new sites into account, the new BVSE map predicts no conduction paths in the SnSn layer and models the neutron (and MD) results much more closely. Indeed, for such an empirical method, the agreement with the conduction paths published by Castiglione *et al.* (2005 ▸) [Fig. 2 ▸(*c*)] is impressive, and gives confidence that the method can be applied to other F^−^ ion conductors containing lone-pair cations.

### 8.2. Calculated conduction barriers for known compounds

To test the wider applicability of the new approach, BVSE maps were calculated for a selection of well characterized fluoride compounds, with the conduction pathways used to estimate the conduction barriers. Table 1 ▸ compares the calculated 1D and 2D conduction barriers with the experimental activation energies and ionic conductivity. In addition to comparison with the conductivity, the calculated barriers were also compared with experimentally determined activation energies.

The table shows a broad correlation between the calculated barriers and the activation energy, including correctly predicting the impressive conducting properties of PbSnF_4_ and eliminating KSnF_3_ and KF as potential conductors. Looking at the two *M*Sn_2_F_5_ compounds, the program correctly predicts that *M* = Na has a higher conduction barrier than *M* = Tl (Battut *et al.*, 1987 ▸), as the sodium compound adopts a crystal structure less suited to ionic diffusion.

Both polymorphs of PbF_2_ show relatively low calculated and experimental activation barriers. However, the resulting conductivity is significantly lower than that of other structures with similar barriers. This is probably a consequence of the smaller number of available vacant sites for anion diffusion within the structure. Whilst the layered structures of the *M*Sn_2_F_5_ and *M*SnF_4_ compounds possess many possible vacant sites for the fluorine ions to jump to, this is not the case for the two binary structures. Significant ionic conductivity within PbF_2_ is only observed at elevated temperatures, facilitated by an increasing concentration of dynamic thermally induced anion Frenkel defects (Bonne & Schoonman, 1977 ▸).

Overall, our benchmarking exercise shows that the program can be used to make a crude prediction of the relative ionic conducting properties of given compounds. The combination of the calculated activation barrier and a visual inspection of the BVSE maps can sort compounds into those that might possess good ionic conductivity and those that probably do not. Whilst this approach cannot be claimed to give an accurate prediction of relative conduction barriers or conductivity for closely related compounds, it has value in providing a threshold value below which diffusion will probably occur. It is, therefore, a quick and simple screening method to identify candidate compounds that are worth exploring experimentally or with more computationally intensive methods.

## 9. Screening for new fluoride ion conductors

**Figure 3:**
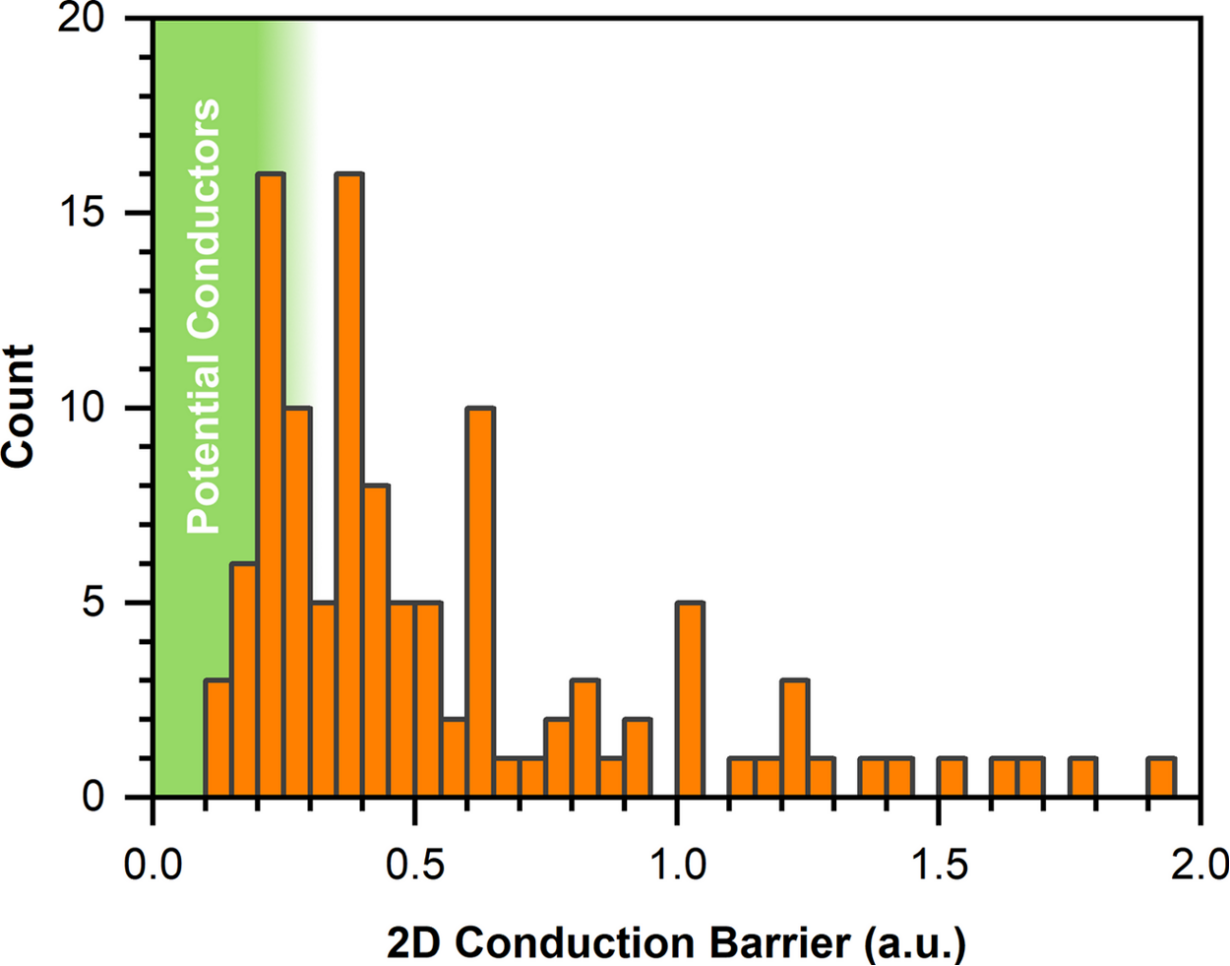
Histogram showing the distribution of conduction barriers observed. Seven compounds were calculated to have barriers larger than 2 a.u. and are not shown in the figure.

A search of the ICSD for fluoride compounds containing at least one of the lone-pair cations Sn^2+^, Pb^2+^, Sb^3+^ and Bi^3+^ identified 136 candidate materials. Fig. 3 ▸ shows a histogram of the calculated 2D conduction barriers and a full list of results can be found in Table 2 ▸. Using the crude benchmarking process described in Section 8.2, we assume that any material with a barrier below 0.2 a.u. has the potential to be a good conductor. However, this is not a hard threshold, and some structures between 0.2 and 0.25 a.u. were also considered to have merit. Overall, it was found that tin compounds were the most likely to have low conduction barriers, whilst only Sn^2+^ and Sb^3+^ tended to have stereoactive lone pairs when coordinated with F^−^.

### 9.1. Tin compounds

Five compounds were identified with a 2D barrier below 0.2 a.u., with an additional nine between 0.2 and 0.25 a.u. The program identified the series *M*Sn_2_F_5_, where *M* = K, Rb or Tl, as being potentially good F^−^ ion conductors. The *M* = Tl case was discussed above in the benchmarking section, whilst the *M* = K and Rb compounds show high conductivities at elevated temperatures. Their structures and ionic conductivities have been characterized previously and are not discussed further here (Battut *et al.*, 1987 ▸; Vilminot *et al.*, 1983 ▸).

**Figure 4:**
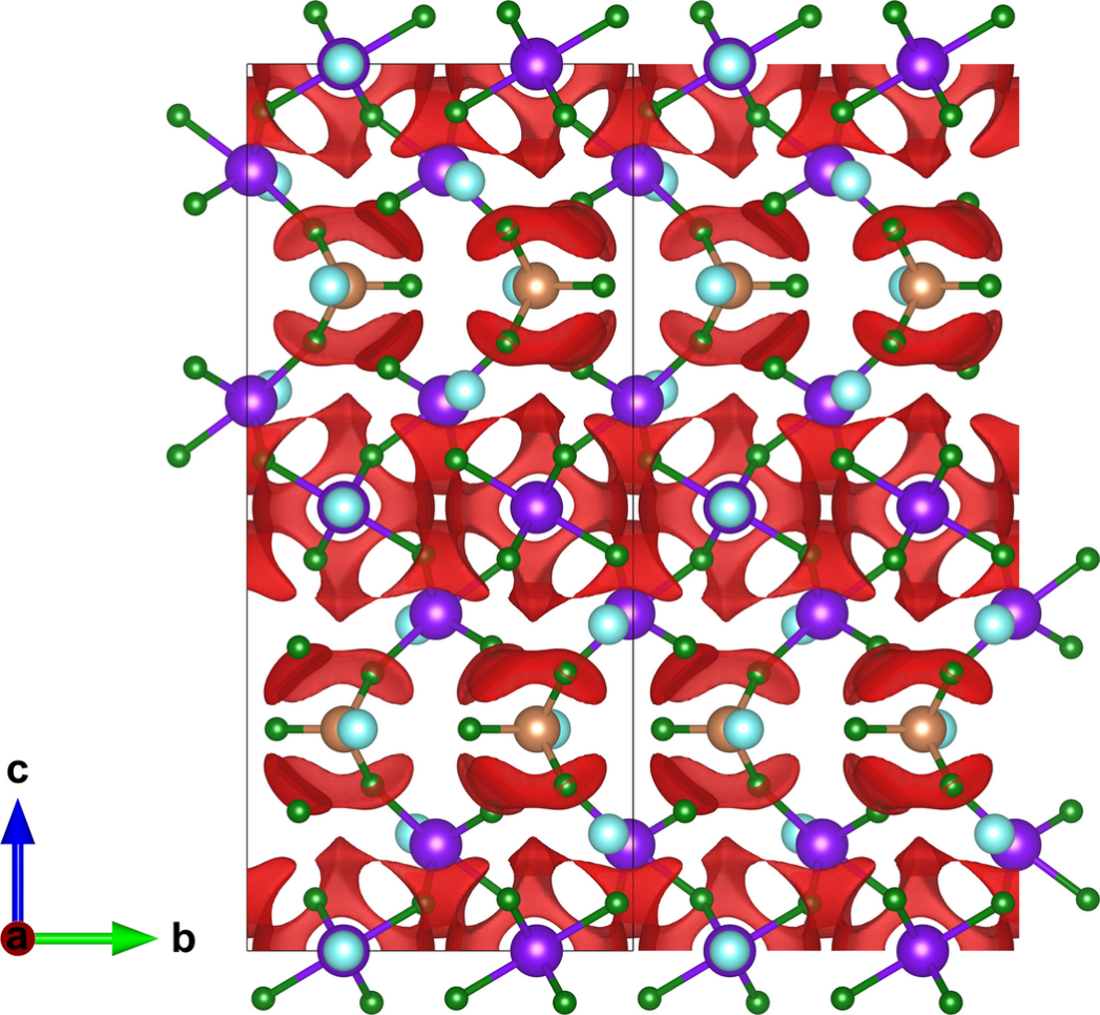
A [100] projection of the SbSn_3_F_9_ crystal structure. Sn^2+^, Sb^3+^ and F^−^ sites are denoted in purple, beige and green, respectively, with lone-pair sites annotated in blue. The red isosurface bounds energies 0.2 units above the minimum energy. Structural data from Kokunov *et al.* (1988 ▸).

The other compound with a 2D barrier below 0.2 a.u. was Sn_3_SbF_9_ (ICSD code 166583; Kokunov *et al.*, 1988 ▸), which adopts an orthorhombic structure in space group *Pbcm*. The structure consists of alternating cation layers, where one layer of Sb^3+^ is followed by three layers of Sn^2+^. The calculated conduction paths, shown in Fig. 4 ▸, indicate that F^−^ conduction takes place within the Sn layers. Unusually, both cations in this structure have been identified as having stereoactive lone pairs. Intuitively, this might be expected to increase the degree of structural disorder within the anion substructure. However, no ionic conductivity data are available for Sn_3_SbF_9_ and only one single-crystal synthesis has been reported (Kokunov *et al.*, 1988 ▸).

Both SnF_2_ and a variety of mixed anion species (with some F^−^ replaced with O2^−^ or Cl^−^) had 2D barriers between 0.2 and 0.25 a.u. An example of this is Sn_4_OF_6_ (ICSD 78356; Abrahams *et al.*, 1994 ▸), which has calculated 2D and 3D barriers of 0.201 a.u. Oxyfluorides have been reported to have moderate conductivity previously, such as LaO_1−*x*_F_1+2*x*_ (Momai *et al.*, 2023 ▸), but no investigation of tin oxyfluorides has been reported.

### 9.2. Lead compounds

**Figure 5:**
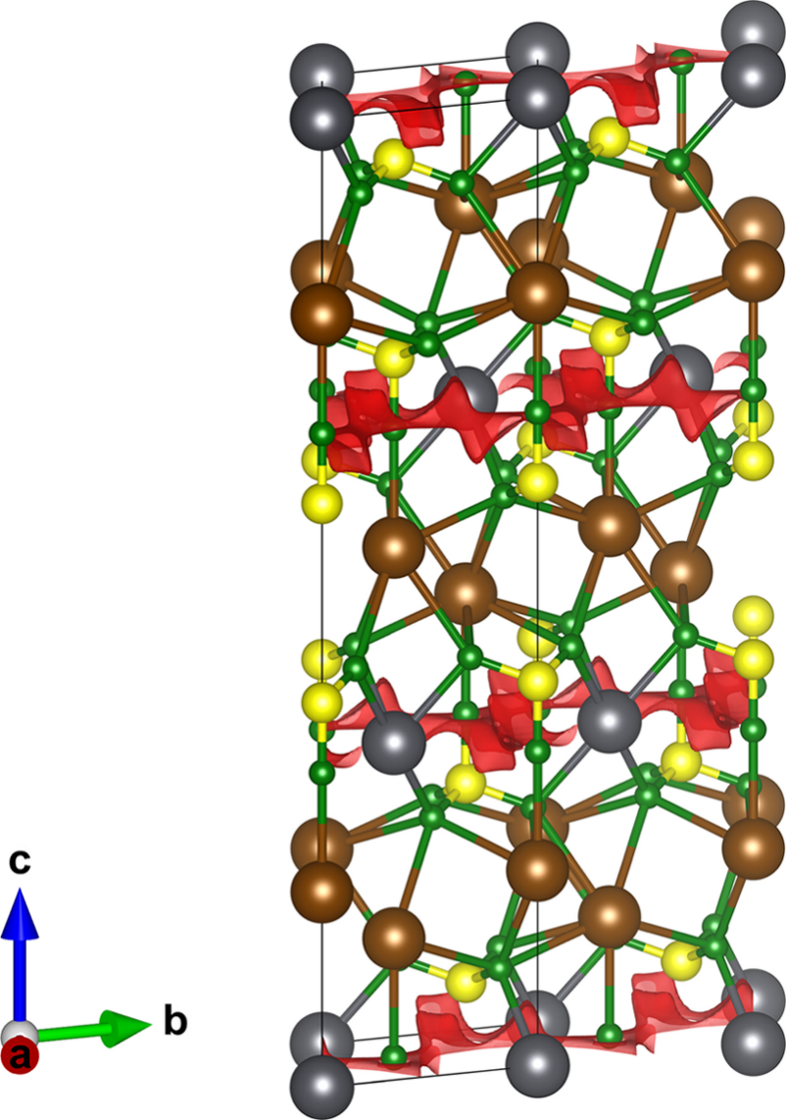
The Tl_2_PbBe_2_F_8_ crystal structure. No lone-pair character was identified. Pb^2+^, Tl^+^, Be^2+^ and F^−^ sites are denoted in grey, brown, yellow and green, respectively. The red isosurface bounds energies 0.2 a.u. above the minimum energy. Structural data from Griesemer *et al.* (2021 ▸).

Only a few Pb-based compounds were identified for further examination. Considerable research effort has been devoted to aliovalent cation doping of PbF_2_, with the ionic conductivity at temperatures close to ambient enhanced by the formation of anion vacancies [*e.g.* in Pb_1−*x*_K_*x*_F_2−*x*_ (Kennedy & Miles, 1976 ▸; Hull *et al.*, 1998 ▸)] or interstitials [*e.g.* in Pb_1−*x*_YF_2+*x*_ (Liang & Joshi, 1975 ▸) and Pb_1−*x*_Zr_*x*_F_2+2*x*_ (Senegas *et al.*, 1986 ▸)]. However, solid solutions of this kind possess a random distribution of the host and dopant cations and cannot be evaluated using the current version of the program. Along with PbF_2_ and PbSnF_4_, whose ionic conductivity has already been discussed, the program identified Tl_2_PbBe_2_F_8_ (ICSD 138579; Griesemer *et al.*, 2021 ▸) as having a barrier below 0.2 a.u. The predicted conduction paths are shown in Fig. 5 ▸. Tl_2_PbBe_2_F_8_ adopts a complex layered structure in space group 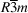. Anion conduction is predicted to take place within the lead–beryllium layers, although no information concerning the ionic conductivity of this material has been reported.

### 9.3. Antimony compounds

**Figure 6:**
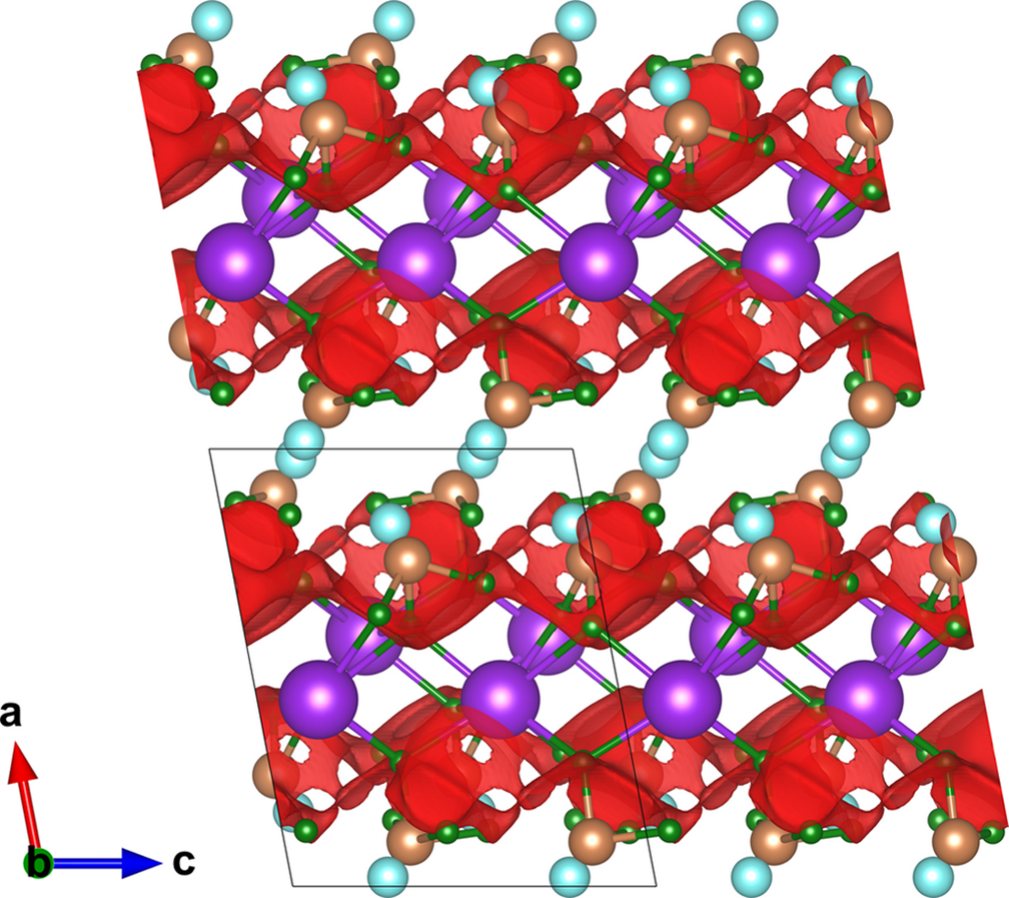
A [010] projection of the KSb_2_F_7_ crystal structure. Sb^3+^, K^+^ and F^−^ sites are denoted with beige, purple and green, respectively, with lone-pair sites annotated in blue. The red isosurface bounds energies 0.2 a.u. above the minimum energy. Structural data from Kavun *et al.* (2005 ▸).

Only two compounds were identified to have conduction barriers below 0.2 a.u., SbF_3_ and KSb_2_F_7_. The latter appears to adopt a rather promising layered structure, with anion diffusion predicted to occur between the potassium–antimony layers (Mastin & Ryan, 1971 ▸). However, unlike PbSnF_4_, the lone pairs are not completely aligned (Fig. 6 ▸). The ionic con­ductivity of KSb_2_F_7_ has been found to be 1.4 × 10^−5^ S cm^−1^ at room temperature, which is moderately high and might be further improved by cation doping to increase the number of F^−^ vacancies (Kavun *et al.*, 2005 ▸). The predicted conduction paths are shown in Fig. 6 ▸. The structure type is not found for any of the other alkali metals; the rubidium structure becomes corrugated due to the larger size of the Rb^+^ cation. Many other stoichiometries are available for alkali metal antimony fluorides, although none have sufficiently low conduction barriers to be considered further. KSbF_4_ is a good conductor, though its conduction appears to be dependent on the creation of anion vacancies (Kawahara *et al.*, 2021 ▸).

### 9.4. Bismuth compounds

**Figure 7:**
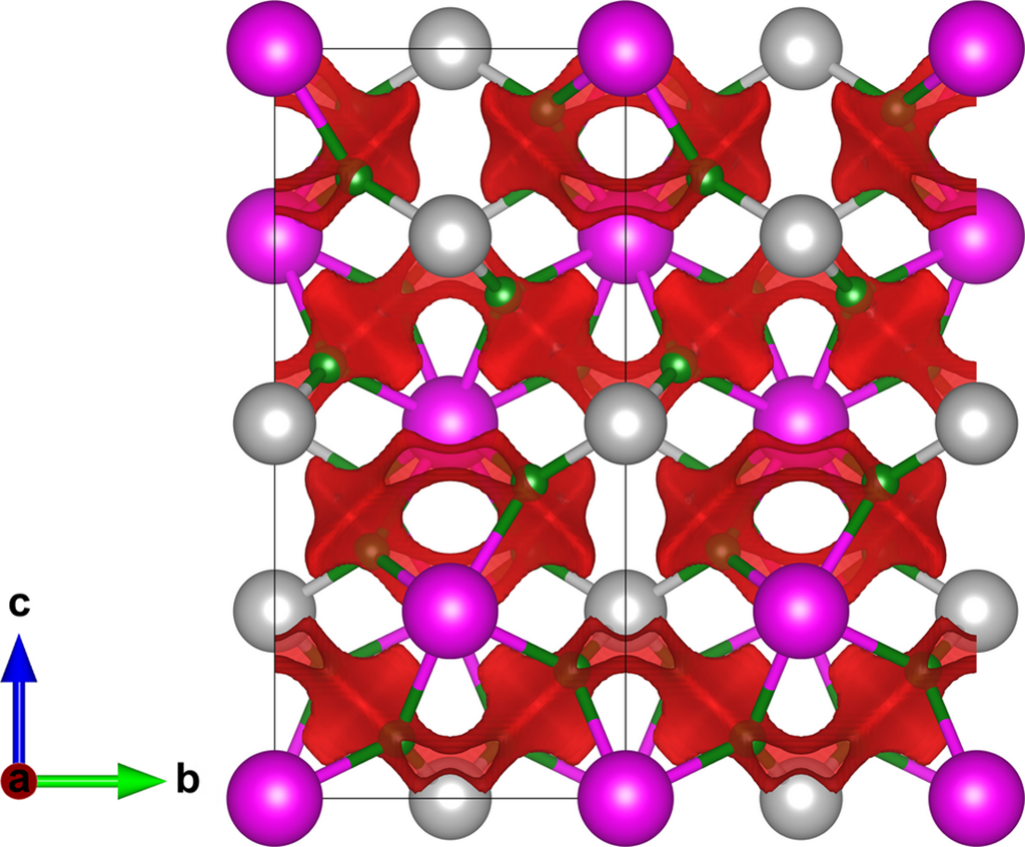
A [100] projection of the BiLiF_4_ crystal structure. No lone-pair character was identified. Bi^3+^, Li^+^ and F^−^ sites are denoted by pink, silver and green, respectively. The red isosurface bounds energies 0.2 a.u. above the minimum energy. Structural data from Schultheiss *et al.* (1987 ▸).

Only 18 distinct structures without substitutional cation disorder were found for Bi^3+^-based fluorides. The highest ionic conductor was identified to be BiF_3_, though the second-best material, BiLiF_4_ (ICSD 65404; Schultheiss *et al.*, 1987 ▸), appears of interest. BiLiF_4_ was found to have a 2D conduction barrier of 0.222 a.u. (Fig. 7 ▸), and its predicted conduction paths are shown in Fig. 7 ▸. This is despite its pure stoichiometric form possessing relatively few vacant sites for anion hopping. An off-stoichiometry or doped material may have potential as a good fluoride ion conductor.

## 10. Discussion

The ionic conductivity of a solid, σ, can be written as 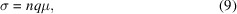
where *n* is the number of charge carriers, which have charge *q* and mobility μ. As the bond valence method focuses only on the last contribution, it cannot as is calculate absolute values of σ [though Wong *et al.* (2021 ▸) combined BVSE with MD to do so].

For mobile ions, a number of factors contribute to μ. These include the structure of the immobile substructure, where high ionic mobility is favoured if the structure contains a large number of suitable vacant sites for the diffusing ions and relatively low energy barriers between them. As shown in this and other work (Meutzner *et al.*, 2019 ▸; Chakraborty *et al.*, 2024 ▸; Kabanova *et al.*, 2024 ▸; Zhang *et al.*, 2020 ▸), the BVSE approach can provide a simple and effective approach to determine both the favoured sites and the migration pathways between them, despite ignoring the role of relaxation of the surrounding counterions during a hop of a mobile ion.

Turning to the properties of the individual ions, smaller mobile ions generally show higher mobility, as they are better able to ‘squeeze’ through gaps formed by the framework of immobile counterions. With its bond valence sum term [equation (3)] and Coulomb-like repulsive term [equation (4)], the expression for the bond valence site energy essentially represents the sum of the ionic sizes and their role in determining the diffusion pathways. However, ionic polarizability effects are also important in promoting easy ionic diffusion within ionic solids, as demonstrated by MD simulations using polarizable ion models [see, for example, the MD studies of PbF_2_ (Castiglione *et al.*, 1999 ▸; Castiglione & Madden, 2001 ▸; Castiglione *et al.*, 2001 ▸)], and these are not included in the bond valence approach. Equally, no account is made for correlated motion of mobile ions, whose study also requires more extensive, and computationally expensive, MD simulations.

Despite the limitations outlined above, the bond valence approach described here offers a quick and simple method to identify candidate fluoride ion conducting materials, providing a motivation to pursue their synthesis. Further studies are required to assess how well it performs for other ionic conducting species (both anionic and cationic) and to what extent the threshold used to predict possible fluoride ion conductors applies more universally. This work is in progress and will be reported in a future publication.

## 11. Conclusions

The demand for new low-cost and environmentally benign technologies to generate and store electrical power is driving extensive research into new ionically conducting materials for applications in fuel cell and battery technologies. The bond valence difference method has been widely used to analyse the diffusion pathways within ionic solids. In this paper, we have presented a modification to the the bond valence site energy calculations to account for lone-pair interactions associated with cation species such as Tl^+^, Sn^2+^, Pb^2+^, Sb^3+^ and Bi^3+^, since these are known to have a profound effect on the anion conduction.

Using the widely studied F^−^ ion conductor PbSnF_4_ as an example, the modified code generates conduction pathways which closely match those found in neutron powder diffraction and MD studies, whilst a comparison between the calculated conduction barriers and experimental studies for a range of fluoride compounds showed a good correlation, especially in the light of the simplicity and swiftness of the approach.

Finally, the method has identified several new candidate materials that are predicted to show high F^−^ ion conductivity.

## Figures and Tables

**Table 1:** Comparison of calculated activation barriers and literature values for activation energy and conductivity Data from Scheiber *et al.* (2022 ▸), Murray *et al.* (2008 ▸), Castiglione *et al.* (2005 ▸), Hull *et al.* (1998 ▸), Foulon *et al.* (1993 ▸), Battut *et al.* (1987 ▸), Vilminot *et al.* (1983 ▸), Vilminot *et al.* (1980 ▸), Bonne & Schoonman (1977 ▸), Boldrini & Loopstra (1967 ▸), McDonald *et al.* (1964 ▸) and Finch & Fordham (1936 ▸).

		Calculated barrier Δ*E* (arbitrary units)			
Structure	ICSD code	1D	2D	Experimental activation energy *E*_a_ (eV)	Log of experimental conductivity at 298 K, log(σ_r.t._) (S cm^−1^)
PbSnF_4_	152949	0.183	0.183	0.31	−1.9
α-PbF_2_	14324	0.168	0.245	0.49	−8.1
β-PbF_2_	86738	0.189	0.189	0.67	−6.2
NaSn_2_F_5_	14136	0.158	0.25	0.88	< −8
TlSn_2_F_5_	38028	0.124	0.124	0.52	−3.2
KSnF_3_	72472	0.225	0.498	–	Negligible
KF	52241	2.863	2.863	–	Negligible

**Table 2:** Calculated conduction barriers from BVSE screening of fluoride compounds (in arbitrary units)

		Conduction barrier (a.u.)		
Formula	ICSD code	1D	2D	3D
(Sn_5_F_9_)(BF_4_)	47140	1.473	1.672	1.851
(Sn_6_F_10_)(TiF_6_)	71234	0.604	0.619	0.619
(SnF_2_)_3_(SbF_3_)	166583	0.124	0.14	0.291
BaSnF_4_	166207	0.358	0.358	0.671
Ca(SnF_3_)_2_	92907	0.228	0.257	0.464
CsSn_2_F_5_	247177	0.22	0.22	0.527
CsSnF_3_	236903	0.338	0.821	0.821
KSn_2_F_5_	99866	0.163	0.163	0.5
KSnF_3_	72472	0.225	0.498	0.565
Na_4_Sn_3_F_10_	9891	0.317	0.32	0.32
NaSn_2_F_5_	14136	0.158	0.25	0.25
Rb(SnF_3_)	72473	0.229	0.524	0.546
RbSn_2_F_5_	247178	0.123	0.242	1.06
Sn(SnOF_5_)	409393	0.565	0.678	1.059
Sn_2_(SnOF_2_)_2_	948	0.187	0.216	0.218
Sn_2_ClF_3_	200032	0.218	0.22	0.22
Sn_2_F_3_BF_4_	15263	1.5	1.795	1.9
SnF_2_	308	0.198	0.201	0.22
Sn_2_F_3_Cl	2088	0.218	0.218	0.218
Sn_2_F_3_I	2419	0.325	0.407	0.76
Sn_3_BrF_5_	200031	0.223	0.248	0.248
Sn_3_F_5_B_4_	15264	0.224	0.224	1.771
Sn_4_OF_6_	78356	0.139	0.201	0.201
Sn_5_Br_4_F_6_	200147	0.291	1.024	1.102
SnClF	647	0.388	0.571	0.765
Tl(Sn_2_F_5_)	38028	0.124	0.124	1.086
Tl(SnF_3_)	72474	0.242	0.506	0.506
PbSnF_4_	152949	0.177	0.177	0.404
[CF_3_SO(OH)_2_](SbF_6_)	262170	4.53	5	5
(SbF_3_)_3_(SbF_5_)	35709	0.334	0.913	1.01
Ba(SbF_5_)	68455	0.247	0.388	0.418
Ba_2_Sb_2_Se_4_F_2_	171429	0.27	0.418	1.142
Cs(Sb_2_F_7_)	14119	0.266	0.388	0.806
CsSbF_3_Cl	28222	0.714	0.714	0.714
CsSbF_4_	201405	0.125	0.48	0.48
K(Sb_2_F_7_)	14118	0.126	0.195	0.473
K(SbF_3_Cl)	4048	0.246	0.289	0.458
α-KSbF_4_	24743	0.168	0.395	0.641
β-KSbF_4_	431489	0.266	0.306	0.479
K_2_SbF_5_	39632	0.19	1.03	1.03
K_2_Sb(P_2_O_7_)F	29323	2.382	2.382	3.748
K_2_SO_4_(SbF_3_)_2_	26438	1.156	1.225	1.436
K_3_(ZrSb_2_F_13_)	123686	0.24	0.38	0.48
K_4_(Sb_2_SnF_14_)	241228	0.459	0.463	0.866
KSb_4_F_13_	4049	0.18	0.376	0.376
KSbClF_3_	20656	0.243	0.284	0.454
KSbF_3_NO_3_	15259	2.459	2.459	5
Li(Sb_2_F_7_)	428176	0.228	0.418	0.418
Mn(SbF_4_)_2_(H_2_O)_2_	62487	0.62	0.62	0.629
Na[F_3_Sb(OH)SbF_3_](H_2_O)	39664	0.221	0.544	0.583
Na(Sb_3_F_10_)	1968	0.26	0.26	0.348
Na(SbF_4_)	24750	0.213	0.279	0.432
Na_2_(SbF_5_)	28061	0.408	0.467	0.467
Na_3_Sb_5_F_18_	60000	0.177	0.364	0.364
NaSbF_3_(NO_3_)(H_2_O)	26393	1.105	1.626	3.5
Ni_3_Sb_4_O_6_F_6_	427047	0.214	0.214	0.214
Rb_2_(SO_4_)(SbF_3_)_2_	64404	1.168	1.177	1.501
RbSbBrF_3_	200109	0.2	0.435	0.539
RbSbF_2_SO_4_	32709	1.079	1.243	1.308
RbSbF_3_Cl	130359	0.255	0.351	0.394
Sb_2_F_4_Cl_5_	2563	1.706	2.313	2.313
Sb_3_O_2_F_5_	67157	0.383	0.397	0.6
Sb_3_Sb_4_F_29_	203183	0.823	1.022	1.532
SbF_3_	16142	0.135	0.136	0.18
SbOF	431209	0.172	0.22	0.273
Sr(SbF_5_)	68454	0.219	0.362	0.383
Sr_2_Sb_2_Se_4_F_2_	171430	0.228	0.228	1.33
TlSbF_4_	201084	0.222	0.229	0.633
Sb_4_F_16_	200035	0.787	0.787	1.132
RbSb_2_F_7_	200574	0.282	0.283	0.71
[Pb(CO_3_)]_2_BaF_2_	280899	2.207	2.207	3.159
[Pb(XeF_2_)_3_](AsF_6_)_2_	391093	1.048	1.244	1.261
[Pb(XeF_2_)_3_](PF_6_)_2_	249570	1.718	1.913	1.92
(PbF_2_)_4_(PbI_2_)	36334	0.206	0.213	1.094
(PbF_2_)_5_(PbI_2_)	36336	0.239	0.243	1.044
(PbF_2_)_7_(PbI_2_)	36337	0.236	0.246	0.944
Cs(PbF_3_)	93439	1.027	1.027	1.027
HfPb_3_F_10_	115823	0.381	0.604	0.61
K_2_Pb(BeF_4_)_2_	9902	0.336	0.336	0.336
KPb(Cr_2_F_9_)	32621	0.425	0.902	0.902
KPrPbF_6_	138755	0.593	0.607	0.62
Pb[AlF_3_(OH)_2_]	79740	1.306	1.415	1.447
Pb(BeF_4_)	24568	0.439	0.622	0.622
Pb(PbF_6_)	23467	0.091	0.358	0.358
Pb(TaF_7_)	417253	0.261	0.607	0.632
Pb(ZrF_6_)	4051	0.395	0.638	0.667
Pb_2_(ZnF_6_)	162074	0.267	0.267	0.573
Pb_2_CrF_7_	174348	0.526	0.542	0.596
PbF_2_(HF)(SbF_6_)	429015	0.64	0.647	0.687
PbF_2_(HF_2_)(PF_6_)	419141	0.826	1.141	1.464
Pb_2_RhF_7_	37141	0.421	0.426	0.488
Pb_3_(Al_2_F_12_)	74861	1.394	1.394	1.63
Pb_3_(AlF_6_)F_3_(H_2_O)	92757	1	1.015	1.396
Pb_3_AlF_9_(H_2_O)	180335	0.781	1.276	2.359
Pb_3_Fe_2_F_12_	67958	0.538	0.596	0.65
Pb_3_ZrF_10_	100600	0.34	0.425	0.425
Pb_5_Al_3_F_19_	91325	0.65	1.525	1.532
Pb_5_Cr_3_F_19_	66050	0.567	0.643	0.653
Pb_5_Ga_3_F_19_	260110	0.415	0.791	0.791
Pb_7_Cl_2_F_12_	10402	0.244	0.401	0.401
Pb_7_F_12_Br_2_	92293	0.242	0.457	0.457
Pb_8_(FeFe_2_F_24_)	88258	0.326	0.804	0.854
PbB_2_O_3_F_2_	263596	2.821	2.821	999
PbB_5_O_7_F_3_	142090	2.461	2.461	2.526
PbBrF	30288	0.371	0.371	0.984
PbCa_2_AlF_9_	180336	0.783	0.854	1.11
PbF(AsF_6_)	411788	0.745	0.831	1.005
PbF(SbF_6_)	429016	0.498	0.614	0.627
α-PbF_2_	14324	0.168	0.245	0.49
β-PbF_2_	250892	0.189	0.189	0.189
PbFBr	5038	0.371	0.371	0.984
PbFI	54755	0.364	0.364	1.349
PbFCl	5037	0.278	0.278	0.98
PbPdF_4_	108992	0.268	0.268	0.479
PbPtF_6_	4057	0.315	0.315	0.317
RbPbF_3_	49591	0.352	0.352	0.352
Tl_2_PbBe_2_F_8_	138579	0.115	0.155	0.55
ZrPb_3_F_10_	115825	0.341	0.428	0.428
Bi_2_F(AuF_4_)_5_	95771	0.65	0.65	0.65
Bi_4_Fe_5_O_13_F	236370	0.438	0.438	0.79
Bi_6_O_7_FCl_3_	1863	0.194	0.968	0.968
Bi_7_F_11_O_5_	167074	0.063	0.252	0.294
BiF_3_	29325	0.13	0.166	0.166
BiLiF_4_	65404	0.148	0.222	0.222
Cs_2_KBiF_6_	9383	1.591	1.591	1.591
Cs_2_NaBiF_6_	9382	1.45	1.45	1.45
Cs_2_RbBiF_6_	9384	1.453	1.453	1.453
Cs_2_TlBiF_6_	9385	1.361	1.361	1.361
Eu_3_Bi_2_S_4_D_4_	230026	0.217	0.217	1.814
KBiF_4_	230026	0.376	0.376	0.376
K_2_BiF_5_	418777	0.303	0.491	0.569
K_6_(BiCl_6_)Cl_2_(H_3_F_4_)	68226	0.257	3.036	3.056
Rb_2_KBiF_6_	9387	0.881	0.881	0.881
Rb_2_NaBiF_6_	9386	0.83	0.83	0.83
RbBiF_4_	63167	0.386	0.386	0.386
SrFBiS_2_	250892	0.207	0.207	1.239

## Data Availability

All the essential data supporting the results reported in this article are available within the article.
